# Squeaking in fourth-generation ceramic-on-ceramic total hip replacement and the relationship with prosthesis brands: meta-analysis and systematic review

**DOI:** 10.1186/s13018-018-0841-y

**Published:** 2018-06-01

**Authors:** Chen-Chen Zhao, Guo-Xin Qu, Shi-Gui Yan, Xun-Zi Cai

**Affiliations:** 1 Department of Orthopaedic Surgery, Second Affiliated Hospital of Zhejiang University School of Medicine, Jiefang Road 88, Hangzhou, 310009 China; 2 Orthopaedic Research Laboratory, Zhejiang University, Jiefang Road 88, Hangzhou, China

**Keywords:** Squeaking, Fourth-generation ceramics, Total hip replacement, Prosthesis brands

## Abstract

**Background:**

Postoperative squeaking in patients who applied the fourth-generation ceramic bearing in primary hip replacement has not been reported systematically; we aim to study the squeaking incidence in the fourth-generation ceramic bearing and related risk factors for squeaking, and we also attempt to explore the relationship between squeaking and prosthetic brands.

**Methods:**

The PubMed, Embase, and Cochrane library were searched, and 14 articles were finally included. Patients’ demographic data, surgical-related information, and prosthesis data were extracted. The occurrence rate of squeaking was calculated by meta-analysis, and subgroup analysis was performed based on prosthetic brands and follow-up time. Regression analysis was further applied to investigate the relationship between various risk factors and squeaking.

**Results:**

The squeaking incidence in patients with the fourth-generation ceramic bearing was 3%. Age, gender, body mass index, and abduction and anteversion angles of acetabular cup might have no influence on squeaking. The squeaking incidence was significantly high with the presence of Delta Motion cup (DePuy, Warsaw, Indiana) and Secure-Fit stem (Stryker, Kalamazoo, MI), and the overall incidence of DePuy femoral stem was relatively small except for the Summit femoral stem. And there was no significant difference of squeaking incidence between less than 5-year and more than or equal to 5-year follow-up subgroups.

**Conclusions:**

In our study, squeaking in the fourth-generation ceramic bearing occurred at a rate of 3%; occurrence rate was high when the Delta Motion cup was applied. We hope for more relevant researches to focus on this issue.

**Electronic supplementary material:**

The online version of this article (10.1186/s13018-018-0841-y) contains supplementary material, which is available to authorized users.

## Background

Ceramic-on-ceramic bearing has been widely applied in total hip replacement for the biological inert, well wearing capacity, and low inflammatory reaction caused by wear particles [1, 2]. The fourth-generation ceramic bearing (BIOLOX Delta, CeramTec AG, Plochingen, Germany) has been used in total hip replacement for many years. Compared with the third generation, it added zirconia (18%), chromium oxide (< 1%), and strontium oxide (< 1%) on the basis of alumina [3]. The addition of these components reduced the risk for prosthesis fracture [4], however, still could not get rid of squeaking [5].

Squeaking was a high-pitched and audible sound, which was considered as a unique but unavoidable phenomenon in hard-on-hard bearing interface [6]. It often appeared in painless or infrequent forms, but their persistence might reduce patient satisfaction, or even cause revision surgery [7]. What is more, it also could be an early sign of pressure peaks indicating incongruency and a risk factor for component breakage [8].

The mechanisms of squeaking were not yet fully understood; prosthesis factors have been regarded as an important contributor [6, 9]. There has been a study comparing four kinds of femoral stems and a finding that the presence of Accolade femoral stem (Stryker, Mahwah, NJ) was significantly associated with hip squeaking [10]. Indeed, different brands of prostheses or different types of prosthesis with the same brand had various designs and materials; we surmised that squeaking incidence might be different when different types of prostheses were used.

To our knowledge, there was no meta-analysis to determine the occurrence rate of squeaking in the fourth generation of ceramic-on-ceramic total hip replacement. We aimed to explore the occurrence rate of squeaking in the fourth generation of ceramic-on-ceramic total hip replacement. The second purpose was to determine the risk factors for squeaking. Moreover, we also attempted to observe the occurrence rate in different prosthetic brands and find the possible relationship between squeaking and prosthetic brands.

## Methods

### Search strategy

This meta-analysis was completed in accordance with the reported guidelines [11]. The PubMed, Embase, and Cochrane library were searched before 22 January 2018 by using the following strategy: “hip and ceramic,” “hip and squeak,” “hip and squeaking,” “hip and noise,” “hip and sound,” “arthroplasty and squeak,” “arthroplasty and squeaking,” “arthroplasty and noise,” “arthroplasty and sound,” “arthroplasty and ceramic,” “squeak and delta,” and “squeaking and delta.” The results were combined, and the repeated publications were excluded; only English language and human studies with full text available were included. When several publications from the same study population were found, the most recent or most detailed one was chosen in our analysis.

### Selection criteria

The title and abstract of the literatures were reviewed by two of the authors (CCZ and GXQ). After a preliminary screening, we read the full text that possibly met the criteria. Studies were included according to the following criteria: (1) underwent primary total hip replacement, (2) applied the fourth-generation ceramic bearing, (3) clearly reported the prosthetic brands, (4) reported squeak or noise as an outcome or complication, and (5) randomized controlled trials and cohort or cross-sectional studies. Literatures which have not specifically point out whether the third or fourth generation of ceramic bearing applied were excluded.

### Data extraction

Three authors (CCZ, GXQ, and XZC) retrieved the included articles and extracted relevant data independently; disagreements were resolved by discussion. Country of study, publication year, total number of patients, number of patients with squeaking, and mean follow-up time were obtained. Moreover, patients’ demographic data (age and gender) and body mass index (BMI) were collected, and in some studies, BMI was indirectly calculated based on weight and height. Surgical information included the abduction and anteversion angles of the acetabular cup which were identified by computerized tomography were also abstracted. And above all, the brands of acetabular cup and femoral stem were extracted.

### Quality assessment

The assessment of the methodological quality of each study was performed with the Consolidated Standards of Reporting Trials checklist [12]. Each study was scored by two of the authors (ZCC and QGX) based on the scale, in which 22 questions were presented and the score was 1 for “Yes” and 0 for “No or unclear.” The disagreements were resolved by discussion.

### Statistical analysis

We used the R software for statistical analysis. The results were presented as weighted mean difference with 95% confidence interval or mean values. Incidence of squeak (*p*) was calculated by total hips (*n*) and the number of hips with squeak (*x*) (if *np* > 5 and *n* (1 − *p*) > 5, then *p* = *x*/*n*, SE (*p*) = √*p* (1 − *p*)/*n*; if *np* < 5 or *n* (1 − *p*) < 5, then *p* = l*n* (*x*/(*n* − *x*)), SE(*p*) = √1/*x* + 1/(*n* − *x*). Heterogeneity between the included studies was evaluated by the *Q* statistic, *τ*^2^, and *I*^2^ statistic [13], and the fixed effect model would be applied if *P* > 0.1 and *I*^2^ < 50%; otherwise, the random effect model would be used (*P* < 0.1 and *I*^2^ > 50%). Sensitivity analysis was further conducted to assess the robustness and reliability of the pooled results. The publication bias was evaluated by funnel plots. All results were presented in the form of forest plots, and *P* values less than 0.05 was considered statistically significant.

## Results

### Search results

We conducted the search based on the mentioned keywords and initially identified 3516 relevant articles. After reviewing the titles and abstracts, 114 articles were further evaluated for eligibility. Then, we found 17 articles might satisfy the inclusion criteria through full text reading, but one did not explicitly report the patients’ number using the fourth-generation ceramics [14], and one got conclusions from the same population [15], and one was a case report [5]. Finally, the remaining 14 articles were included for meta-analyses [16–29]. The flowchart of literature selection was shown in Additional file 1: Figure S1.

### Incidence of squeaking

**Fig. 1:**
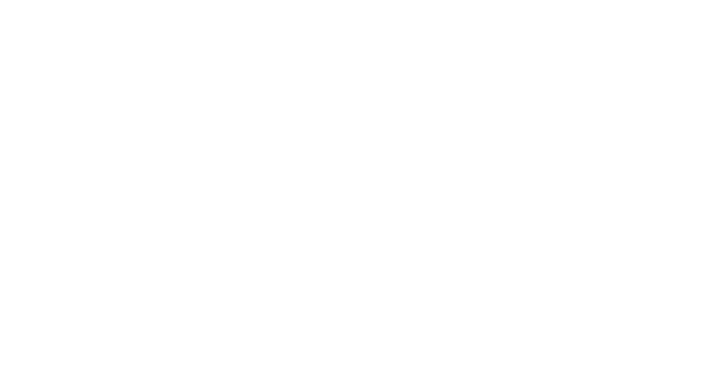
Forest plots for squeaking incidence of all included studies. *I*^2^, *τ*^2^, and *P* value are tested for heterogeneity

**Fig. 2:**
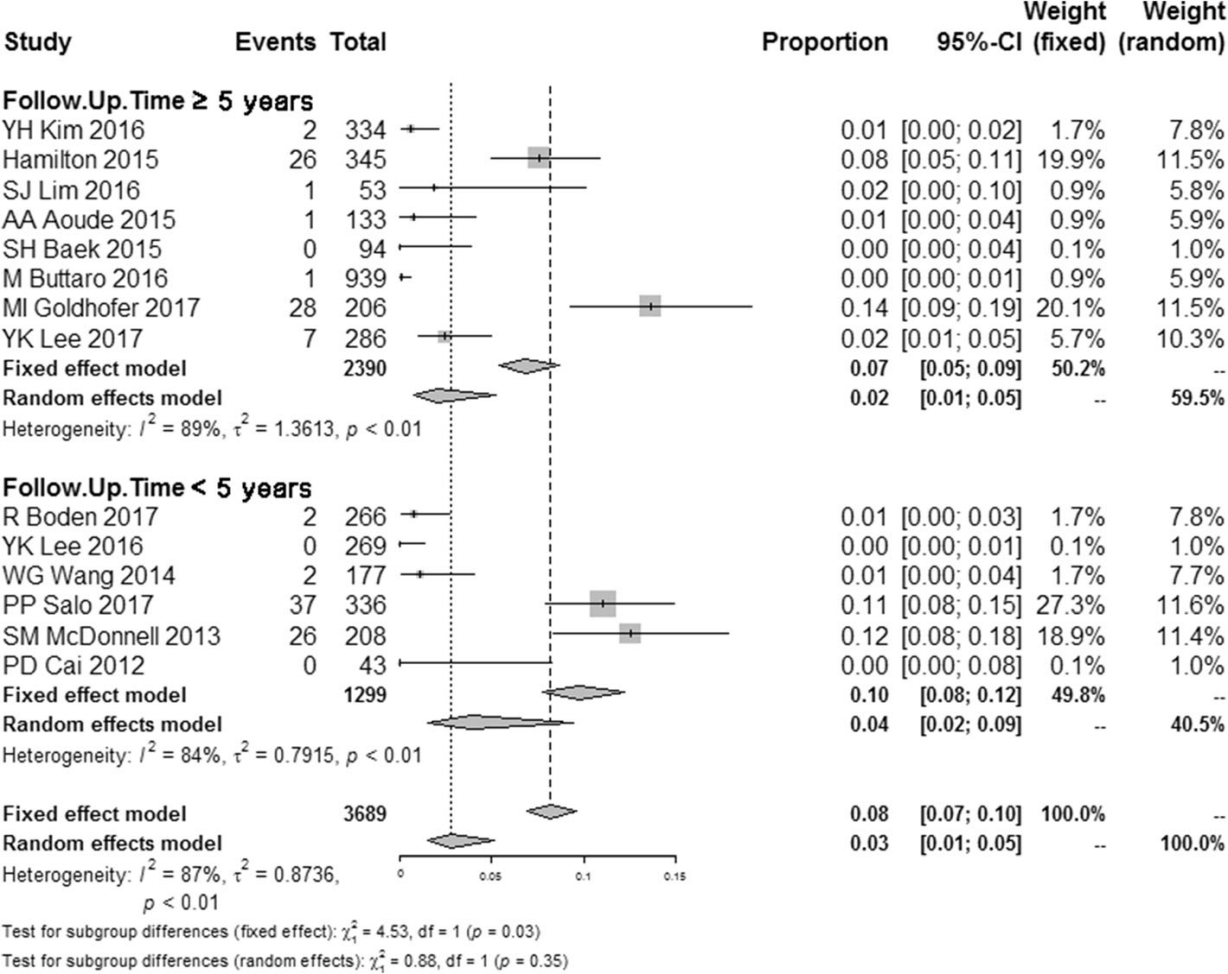
Forest plots for squeaking incidence in less than 5-year and more than or equal to 5-year follow up groups. *I*^2^, *τ*^2^, and *P* value are tested for heterogeneity

The demographic analysis showed that the mean age of all included cases was 53.35 years, and the mean BMI was 26.73 kg/m^2^ (Table 1). A total of 133 cases reported squeaking, and we finally calculated the squeaking incidence nearly to be 3% (*I*^2^ = 87%) (Fig. 1). We also did regression analysis to establish the influence of patient factors and surgical factors on squeaking. Results showed that age (*P* = 0.50), gender (*P* = 0.73), BMI (*P* = 0.46), and abduction (*P* = 0.61) and anteversion angles (*P* = 0.81) of acetabular cup had no significant effect on the occurrence of squeaking (Table 2). Moreover, we divided the studies into two subgroups based on follow-up time (less than 5-year follow-up group, more than or equal to 5-year follow-up group). We revealed that squeaking incidence has no significant difference between the two subgroups (*P* = 0.35, Fig. 2). In addition, 32 patients suffered from postoperative dislocation; the rate of dislocation was approximately 1%.

**Table 1:** Studies included in the meta-analysis

Author	Year	Country	Total hips	Patients with squeak	Acetabular cup	Femoral stem	Mean follow-up time (months)
Cai et al.	2012	China	43	0	NA	NA	39.7
McDonnell et al.	2013	Australia	208	26	Delta Motion (DePuy)	Finsbury Type C (Finsbury Orthopaedics)/SL Plus MIA stem (Smith & Nephew)/Tri-Lock and Corail (DePuy)	21
Wang et al.	2014	China	177	2	Pinnacle (DePuy)	Corail and Summit (DePuy)	12
Hamilton et al.	2015	Canada	345	26	Pinnacle (DePuy)	AML, Prodigy, Summit, Srom, and Corail (DePuy)	64
Aoude et al.	2015	Canada	133	1	Pinnacle (DePuy)	Corail, SROM, Summit, Trilock and Prodigy (DePuy)	72
Baek et al.	2015	Korea	94	0	Bencox (Corentec)	Bencox (Corentec)	60
Kim et al.	2016	Korea	334	2	Pinnacle (DePuy)	Proxima (DePuy)	157.2
Lee et al.	2016	Korea	269	0	Exceed eABT™ (Biomet)	Taperloc (Biomet)	24
Lim et al.	2016	Korea	53	1	Bencox (Corentec)	Bencox (Corentec)	64
Buttaro et al.	2016	Argentina	939	1	Pinnacle (DePuy)/Combi (Wlink)/Trinity (Corin)	Corail, SROM and Cstem (DePuy)/CFP (Wlink)/Minihip (Corin)	64
Boden et al.	2017	UK	266	2	Pinnacle (DePuy)	Corail (DePuy)	18
Salo et al.	2017	Finland	336	37	Continuum (Zimmer)/Exceed (Biomet)/Pinnacle (DePuy)	ML Taper (Zimmer)/Bimetric (Biomet)/Corail, Srom, and Summit (DePuy)	25.6
Goldhofer et al.	2017	Australia	206	15	Delta Motion (DePuy)	Secur-Fit stem (Stryker)	60
Lee et al.	2017	Korea	286	7	Pinnacle (DePuy)	Proxima (DePuy)	66.5

**Table 2:** Meta-regression analysis for the factors affecting the squeaking incidence

Variables	Total hip	Study	*P* value	95% CI
Age	3689	14	0.498	− 0.430 to 0.223
Gender	3689	14	0.727	− 15.57 to 21.62
BMI	2145	10	0.464	− 1.935 to 0.967
Abduction	1537	7	0.606	− 0.889 to 1.415
Anteversion	1494	6	0.817	− 1.309 to 1.584

*BMI* body mass index, *CI* confidence interval

### Squeaking and prosthetic brands

**Fig. 3:**
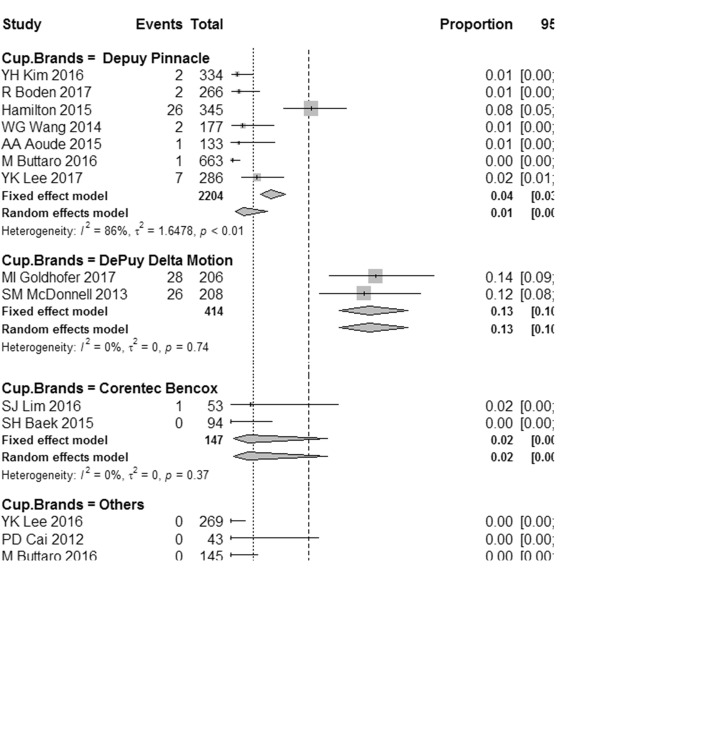
Forest plots for squeaking incidence in each subgroup based on acetabular cup brands. *I*^2^, *τ*^2^, and *P* value are tested for heterogeneity

We performed subgroup analysis based on the prosthetic brands to study its impact on squeaking. Firstly, we analyzed for the acetabular brands’ effect. We found that the occurrence rate of squeaking in Delta Motion group was as high as 13%, significantly higher than that in the other three groups (*P* < 0.001). The squeaking incidence was 2% in Bencox group and 1% in Pinnacle group. Additionally, there was no squeaking case reported when other brands of acetabular cup (Link, Biomet, Wlink, and Corin) were applied. The results were shown in Fig. 3.

**Fig. 4:**
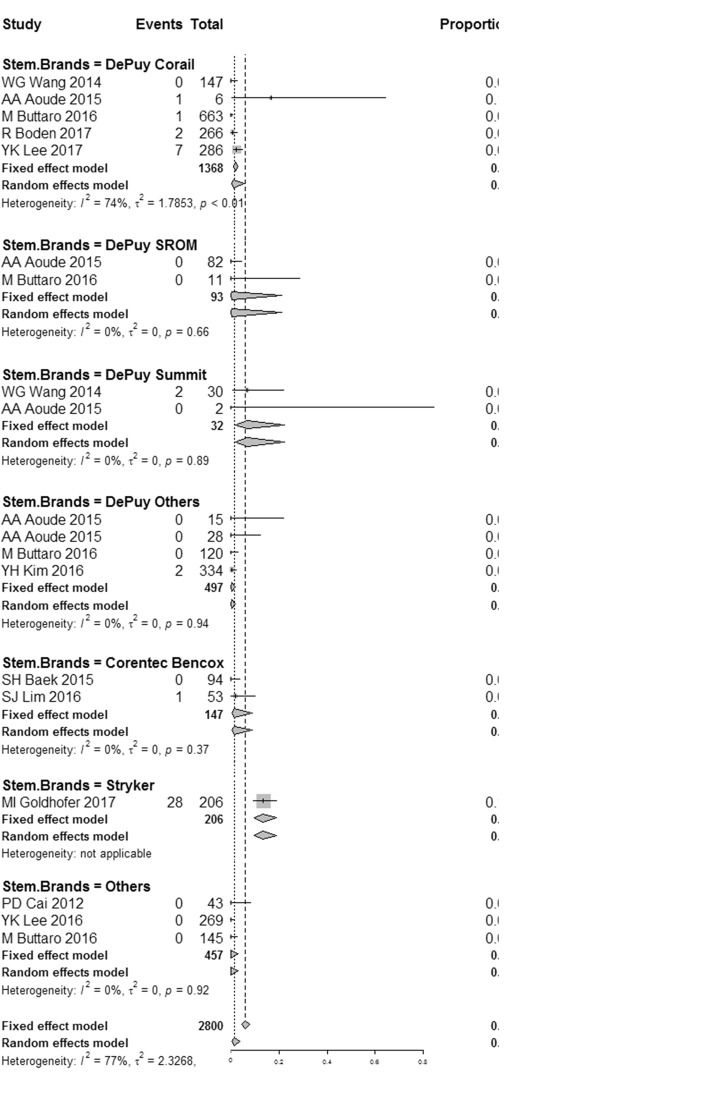
Forest plots for squeaking incidence in each subgroup based on femoral stem brands. *I*^2^, *τ*^2^, and *P* value are tested for heterogeneity

We then analyzed for the femoral stem brands effect. Results showed that the occurrence rate of squeaking with the presence of Secure-Fit stem was 14%, significantly higher than that with the other brands of stems (*P* < 0.001). Among patients applying the DePuy stems, the squeak incidence was 7% with the DePuy Summit stem, but only 1% with the DePuy Corail stem, and no squeaking case was reported with the DePuy Srom stem. The occurrence rate was 2% with the presence of Bencox stem; no squeak case was reported when the other brands of stems (Link, Wlink, and Corin) were applied (Fig. 4).

### Quality assessment and publication bias

Most of the included studies had a moderate score in the methodological quality assessment, and the mean score was 14.3 (10–18), which meant the included studies were of relative moderate quality. Although funnel plots showed a certain degree of publication bias, the sensitivity analysis demonstrated that our pooled results were robust and reliable (Additional file 2: Figure S2).

## Discussion

We did the first meta-analysis to study the squeaking incidence in the fourth-generation ceramic bearing. We found that the occurrence rate of squeaking in patients who applied the fourth-generation ceramic bearing in primary total hip replacement was nearly 3%. The presence of DePuy Delta Motion cup significantly increased the incidence of squeaking, and so did the Secure-Fit femoral stem. Age, gender, BMI, and anteversion and abduction angles of acetabular cup were not found to be associated with squeaking. There was no significant difference of the squeaking incidence between less than 5-year and more than or equal to 5-year follow-up subgroups.

The fourth-generation ceramic bearing was first applied in clinical at 2000 [28]; Yang et al. once proposed that improvement of material properties may reduce the risk for squeaking [30], and there was indeed no report of squeak in the fourth-generation ceramic bearing in the early study [31], but Buttaro et al. presented the first case who suffered from postoperative squeaking in 2012 [5]. Since then, more and more researches began to declare squeaking as a complication of the fourth-generation ceramic bearing. Thus, we did this systematical study and found the occurrence rate was 3%. Previous study has shown that the occurrence rate in the third-generation ceramic bearing fluctuated at 0.7–20.9% [32]. Stanat and Capozzi conducted the meta-analysis for the studies which mainly applied the third-generation ceramic bearing and reported the squeaking incidence was 2.4% [33]. It seems that the fourth-generation ceramic bearing does not have a significant superiority in postoperative squeaking.

The mechanisms of postoperative squeaking have not been clearly elucidated at present; many theories have been put forward with the desire to make a comprehensive explanation, including edge loading, lubrication imbalance, rim impingement, micro-separation, and stripe wear [6, 7]. The prosthetic factors were accepted to be an important part; the designs and materials were different in various brands of prosthesis, or in the same brand but different types of prosthesis [9, 34]. For instance, the Stryker Trident PSL cup had an elevated rim to increase the ceramic stabilization but caused more squeaking for impingement between femoral neck and cup [35]; the design of Delta Motion cup did not permit reinforcement by auxiliary screws, which might impact implant stability under specific conditions and be a potential risk for squeaking [36]. Moreover, surgical techniques were connected to squeaking. Incorrect acetabular cup orientation could cause rim impingement and edge loading [37]. The patient’s factors also counted; reports showed that younger, taller, and more active patients may be easier to suffer hip squeaking [38]. And preoperative diagnosis of rheumatoid arthritis might be a risk factor for squeaking [10].

In our study, the presence of DePuy Delta Motion cup significantly increased squeak. Delta Motion was a pre-assembled cup with large-diameter femoral heads, which was engineered to maximize head-neck ratios and reduce the risk for impingement, thus decreased the possibility of squeak [20]. However, it did not achieve the intended purpose. In fact, the technology advancement of the fourth-generation ceramic reduced the thickness of bearing and promoted the application of large-diameter femoral heads. Large-diameter femoral heads could reduce joint instability and give patients a similar sense of movement to healthy subjects [39, 40], which has become increasingly popular in hip replacement. However, it might cause micro-separation during movement because of the small opening angle between femoral head and acetabular cup [41]. Moreover, if the acetabular cup was too vertical, large femoral head would significantly increase the load on its edge [42]. Micro-separation and edge loading are two critical mechanisms for squeaking.

Moreover, we know that squeak originates from irregular vibrations, which are resulted from the combined effects of initial pulse, vibration propagation, and amplification [43]. The DePuy Delta Motion cup with larger femoral head might significantly increase the articulation surface and frictional moment at the inadequately lubricated condition, thus inducing vibration [44]. And they might increase the prosthesis mass, which could reduce the natural frequency of vibration, increase the amplitude, and further amplify the initial vibration [15]. In addition, McDonnell pointed out that soft tissue laxity and a wider range of movement may provide supportive environment for squeaking among patients applying Delta Motion [20]. Combination of the above effects might be responsible for the significant higher incidence of squeak in Delta Motion cup.

Femoral stem design and metallurgical technology also have a great impact on squeak. Studies have reported that the Stryker Accolade femoral stem was prone to generate squeak [10, 33]. In the fourth-generation ceramic bearing, only one study reported the application of Stryker femoral stem (Secure-Fit); it had the highest incidence of squeak in all the used stems, and this may be related to the unique design of Stryker femoral stem (high rim and short neck) [9]. But Secure-Fit femoral stem had a relatively wider neck with thick long stem compared to the Accolade femoral stem [6, 15], and it was made of Ti-6Al-4V, which has lower tensile properties and flexibility than Ti-12Mo-6Zr-2Fe (Accolade femoral stem), so it was not conducive to adhere to the femur, resulting abrasion and lubrication imbalance and then causing squeak [35, 45]. Therefore, we speculated it was highly possible that the high incidence was attributed to the combinative use of Delta Motion cup, but the specific link need to be further explored.

In addition, the occurrence rate of squeak with the presence of Summit stem was high, but the applied patients’ number was small; our conclusion still required further verification.

We also conducted regression analysis for age, gender, BMI, acetabular cup abduction and anteversion angle; these factors have no significance for squeak, and the results were consistent with previous studies [33, 46]. Lee [46] once pointed out that abduction angle of acetabular cup was a risk factor for squeak, but his research was limited to the Asian population, different from ours.

Furthermore, dislocation, exposure types, and surgical indications might have effects on squeaking. Dislocation often had similar risk factors for squeaking, including improperly placed prosthesis, unreasonable prosthesis design, and soft tissue laxity. Excessive or insufficient anteversion of acetabular cup were important reasons for dislocation [47], which might not only cause an increasing load on the cup edge, but also lead to rim impingement. In our included studies, posterolateral, posterior, direct lateral, and anterolateral approaches were applied in the surgery. Different surgical approaches had different effects on soft tissue and affect joint stability [48], which might induce squeaking. As for surgical indications, study has shown that rheumatoid arthritis was related with squeaking [10]. Different indications had different surgical requirements, and patients’ basic conditions were also different, which might have potential effects on squeaking. Our research was limited to the factors such as data amounts and the designs of the study; it was difficult to make further assessment. In the future, clinical data based on large sample sizes and multi-centers can help to establish the specific effects of these factors on squeaking.

The high heterogeneity was a limitation of our meta-analysis. We found the high heterogeneity mainly distributed in studies applied the DePuy Pinnacle cup and the DePuy Corail femoral stem. These two prostheses are currently most widely used; studies from different regions and populations were included in our analysis, while studies that applied other types of prostheses almost came from the same countries and populations. Thus, we supposed the high heterogeneity may be due to this. Our research still has some other limitations. Firstly, studies included were mainly on the issue of squeaking, and the data was all retrieved from the published literatures; these might cause selection or publication bias, and strictly designed and high-quality RCTs are needed in the future. Secondly, some available articles applied a variety of brands of prostheses but did not report the corresponding brands of prostheses with squeaking; removing these literatures during subgroup analysis might have an influence on results. Thirdly, the reported studies about squeaking generated in the fourth-generation ceramic bearing is limited; our findings require to be further supported by larger number of cases and more long-term follow-up studies.

## Conclusion

In conclusion, the overall incidence of squeaking in the fourth-generation ceramic bearing was nearly 3% in our study. Among the prostheses we studied, the occurrence rate was highest with the presence of Delta Motion cup, which might be attributed to the increased frictional moment and induction and amplification of initial vibration. Age, gender, BMI, and other related factors might have no significant effect on squeaking in the fourth-generation ceramic bearing. Finally, we look forward to more relevant researches focusing on this issue.

## Additional files

Additional file 1: **Figure S1.** Flowchart of literature selection. (TIF 701 kb)

Additional file 2: **Figure S2.** Sensitivity analysis of the study. (TIF 340 kb)

## Abbreviation

BMI: Body mass index
