# Supplementary figures and images for: Squeaking in fourth-generation ceramic-on-ceramic total hip replacement and the relationship with prosthesis brands: meta-analysis and systematic review
Source: J Orthop Surg Res. 2018 Jun 1;13:133. doi: 10.1186/s13018-018-0841-y (PMC5984797; doi:10.1186/s13018-018-0841-y)

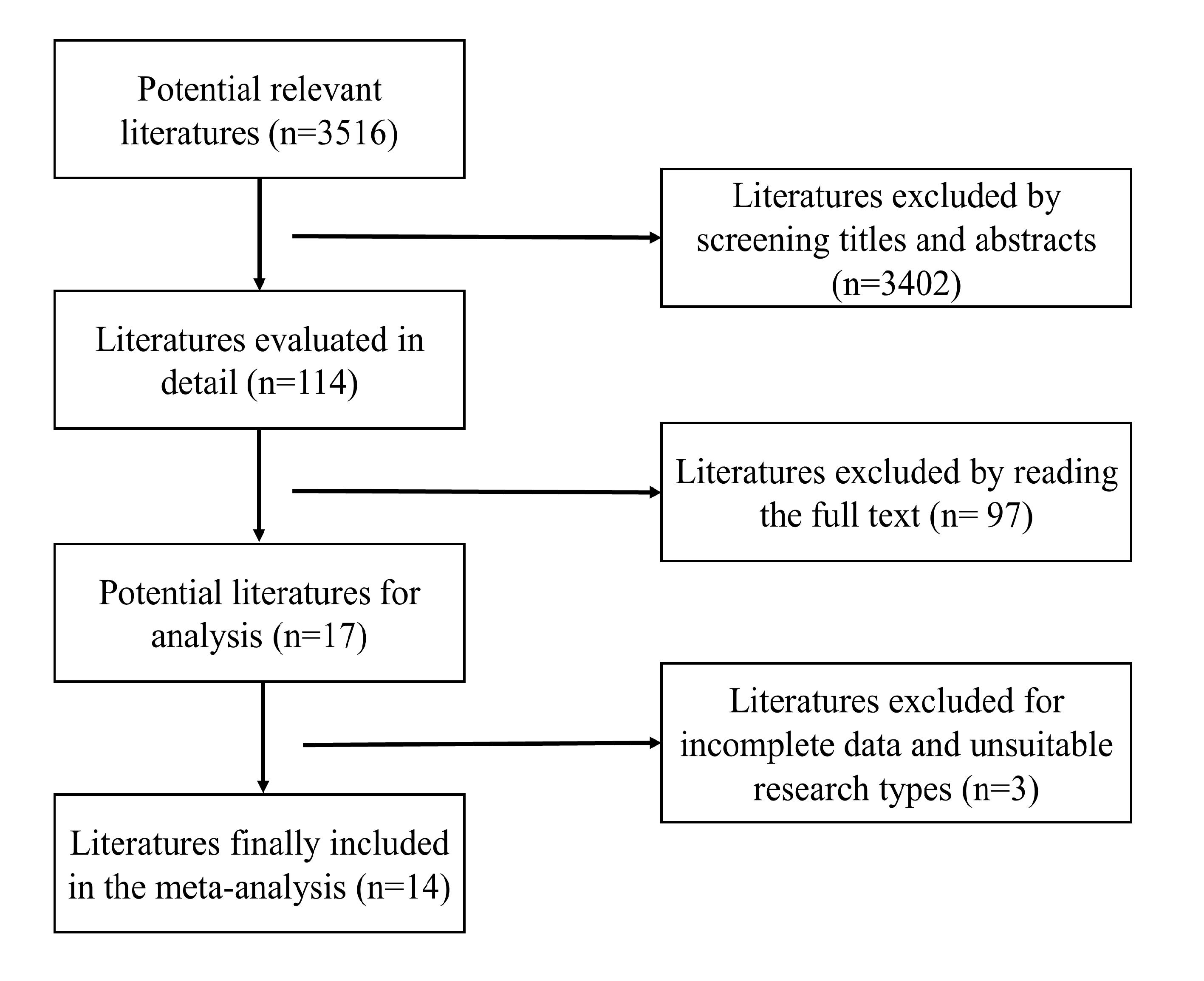

Supplement: Supplementary file 1 — Figure S1. Flowchart of literature selection. (TIF 701 kb) [file 13018_2018_841_MOESM1_ESM.tif]

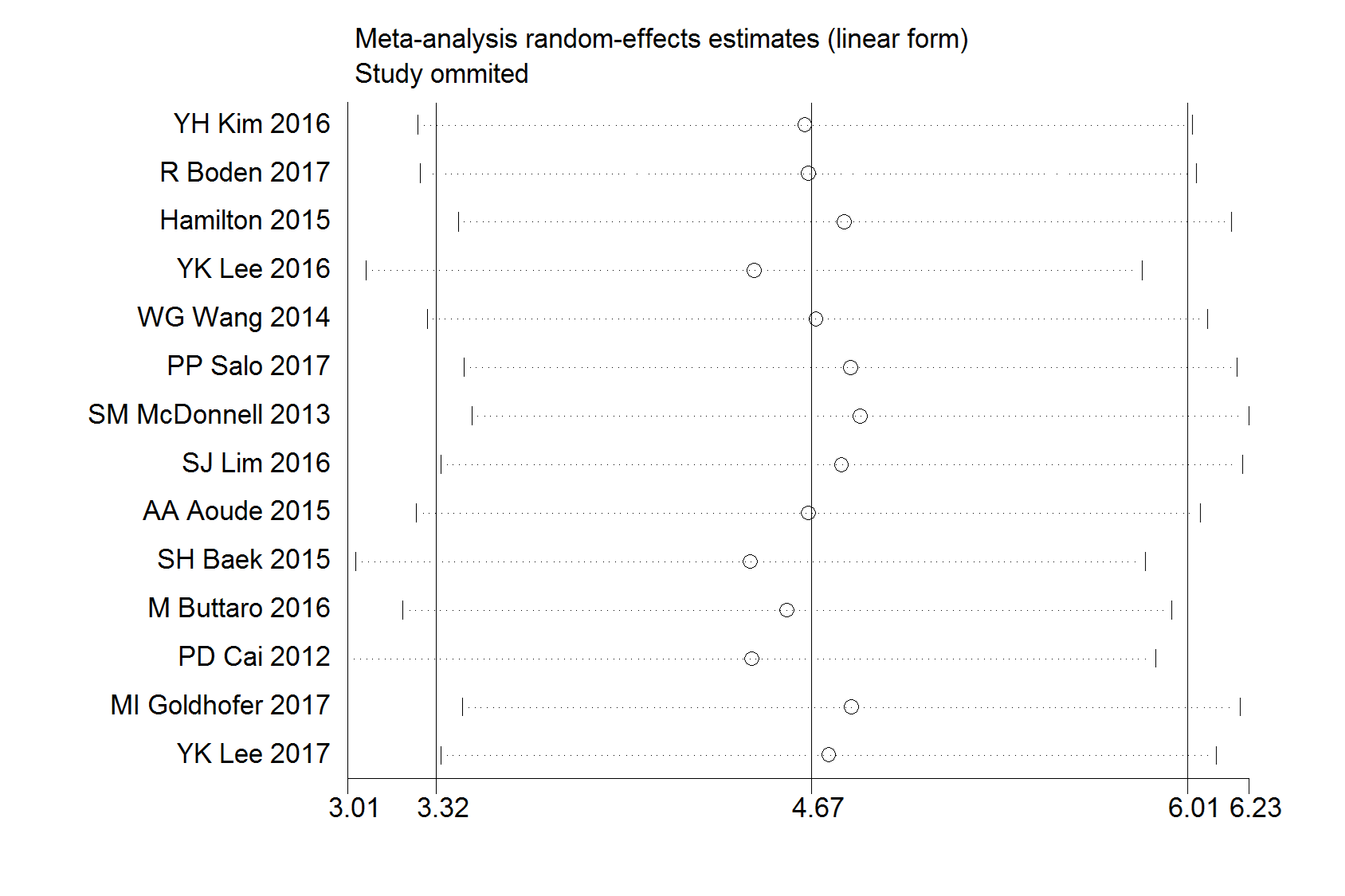

Supplement: Supplementary file 2 — Figure S2. Sensitivity analysis of the study. (TIF 340 kb) [file 13018_2018_841_MOESM2_ESM.tif]
